# Comparative Efficacy of Xianling Gubao Capsules in Improving Bone Mineral Density in Postmenopausal Osteoporosis: A Network Meta-Analysis

**DOI:** 10.3389/fendo.2022.839885

**Published:** 2022-02-18

**Authors:** Ming-hui Luo, Jin-long Zhao, Nan-jun Xu, Xiao Xiao, Wen-xuan Feng, Zi-ping Li, Ling-feng Zeng

**Affiliations:** ^1^ The 2nd Affiliated Hospital of Guangzhou University of Chinese Medicine, Guangzhou, China; ^2^ The Second Clinical Medical College of Guangzhou University of Chinese Medicine, Guangzhou, China

**Keywords:** traditional Chinese medicine, efficacy, Xianling Gubao capsule, postmenopausal osteoporosis, network meta-analysis

## Abstract

**Objective:**

The clinical efficacy of Xianling Gubao capsule (XLGB) and its combination therapy in the treatment of postmenopausal osteoporosis (PMOP) was systematically evaluated by frequency-based network meta-analysis.

**Methods:**

We searched the China National Knowledge Infrastructure (CNKI), Wanfang, SinoMed, PubMed, Embase and Cochrane Library databases to identify clinical trials of XLGB for the treatment of PMOP from the establishment of each database to November 22, 2021. The quality of the included studies was evaluated by using the risk of bias assessment tool version 2.0 (Rob 2.0) recommended by Cochrane. Stata 14.0 was applied for statistical analysis of the data, and the surface under the cumulative ranking curve (SUCRA) was used to rank the intervention measures of each outcome index.

**Results:**

This study included 22 clinical trials (including 19 RCTs and 3 non-RCTs) involving 12 drug therapies. According to the results of the network meta-analysis and SUCRA, the best three interventions for improving lumbar bone mineral density (BMD) are XLGB+BP+calcium (83.7%), XLGB+BP (68.5.7%) and XLGB+VD (67.1%). XLGB+calcium was the best combination regimen for improving femoral neck BMD and increasing bone Gla protein (BGP) and alkaline phosphatase (ALP) contents in serum. The SUCRA values of XLGB+calcium for improving the three outcome indicators were 68.0%, 59.5% and 82.1%, respectively.

**Conclusions:**

The results of this network meta-analysis show that combined application of XLGB can effectively improve BMD and serum BGP and ALP compared to calcium alone, VD or BP. In the future, multicenter, large-sample and double-blind clinical RCTs should be carried out to supplement and verify the results of this study.

## Introduction

Postmenopausal osteoporosis (PMOP) is a systemic metabolic bone disease characterized by decreased bone mass, increased bone fragility and easy fracture (1). PMOP generally occurs within 5 to 10 years after menopause (2). Based on epidemiological statistics, there are approximately 27.6 million patients with osteoporosis over 50 years old in EU countries, including 22 million women, with a prevalence rate of 22.1% (3). The prevalence rate of osteoporosis in women is approximately three times that in men (3). The decline in ovarian function and the decrease in estrogen secretion in postmenopausal women can lead to an imbalance in bone turnover, a decrease in bone mass and an increase in bone fragility (4). A low BMI index, low milk intake, alcohol consumption, fracture history and smoking are risk factors for PMOP, and strengthening the management of the above controllable risk factors is beneficial to reduce PMOP complications (5). At present, the main drug used for the treatment of PMOP is a bone resorption inhibitor. Bisphosphonates, estrogens and other drugs recommended in clinical practice guidelines have good clinical efficacy, but their safety remains a concern (4, 6). Therefore, it is important to seek effective drugs while improving drug safety.

In traditional Chinese medicine, osteoporosis belongs to the category of “*Guwei*”, which is closely related to kidney deficiency: “The kidney stores essence and the main bone generates marrow” (7). Therefore, kidney nourishing is closely related to bone marrow regeneration. Xianling Gubao capsule (XLGB) is mainly used to clinically treat osteoporosis and osteoarthritis. XLGB includes *Epimedium*, *Dipsacus*, *Psoralea*, *Rehmannia glutinosa*, *Salvia miltiorrhiza* and *Anemarrhena asphodeloides*, which have the functions of nourishing the liver and kidney, activating blood circulation and dredging collaterals, and strengthening tendons and bones (7, 8). Kidney-tonifying herbs or compounds, such as XLGB, can regulate the proliferation and differentiation of osteoblasts and osteoclasts through a variety of signaling pathways to achieve a dynamic balance (8). XLGB is widely used in the treatment of PMOP in China, but compared with other types of drug regimens or combination therapy, an evidence-based foundation is lacking. Although XLGB is widely used in China, the comparison of efficacy and safety between XLGB and other drugs or combination therapies remains controversial (7, 8), and it is necessary to further evaluate efficacy between XLGB and other therapies. Therefore, this study used the frequency network meta-analysis method to compare the clinical efficacy of XLGB and other drug regimens for the treatment of PMOP to provide an evidence-based foundation for the rational clinical application of XLGB.

## Materials and Methods

### Inclusion Criteria

The inclusion criteria were as follows. 1) Participants: female postmenopausal patients with a clear diagnosis of primary osteoporosis and clear diagnostic criteria for osteoporosis in the study. 2) Interventions and comparisons: the experimental group was treated with XLGB alone or combined with XLGB on the basis of routine intervention measures in the control group; the control group was treated with no intervention, placebo or conventional Western medicine. 3) Outcome measure: the main outcome indicators were lumbar bone mineral density (BMD) and femoral neck BMD, and secondary outcome indicators were bone Gla protein (BGP) and alkaline phosphatase (ALP); all included studies involved at least one of the above evaluation indicators. 4) Study design: randomized controlled trials (RCTs) and nonrandomized controlled trials (non-RCTs). 5) To ensure the test efficiency of this study, the number of studies included in the intervention measures was ≥ 2.

### Exclusion Criteria

The exclusion criteria were as follows: 1) animal experiments and case reports; 2) studies with incorrect or incomplete data; 3) studies with unobtainable full texts; and 4) repeated published literature.

### Literature Search

We searched the China National Knowledge Infrastructure (CNKI), Wanfang, SinoMed, PubMed, Embase and Cochrane Library databases to identify clinical trials of XLGB for the treatment of PMOP. The retrieval time limit was from the establishment of each database to November 22, 2021. Each database was searched using subject words combined with free words. English search terms included “Xianlingguao”, “Xianling Gubao”, “XLGB”, “osteoporosis”, “postmenopausal osteoporosis”, “postmenopausal osteoporosis”, “primary osteoporosis”, and “PMOP”. The retrieval strategy for each database is shown in **Supplementary Material 1**.

### Literature Screening and Data Extraction

Two researchers followed the inclusion and exclusion criteria to independently screen the literature and extract data; any disagreements were resolved by the corresponding author. The extracted contents included basic information, bias risk factors, and outcome index data.

### Bias Risk Assessment

Literature quality was evaluated using Cochrane’s recommended risk of bias assessment tool version 2.0 (Rob 2.0) (9). The evaluation tool assesses the risk of bias in five areas, including bias generated in the random process, bias deviating from the established intervention, bias of missing outcome data, bias of outcome measurement and bias of selective reporting of results.

### Statistical Analysis

The relative advantages and disadvantages of network meta-analysis can be evaluated by direct or indirect comparisons and the cumulative ranking probability. This study was based on the frequency framework and used Stata/MP 14.0 with the mvmeta and network packages (10–12). All indexes were analyzed using a random effect model. As the outcome indicators considered in this study were continuous variables, the mean difference (MD) was used as the effect value; the result is expressed with a 95% confidence interval (CI).

The size of the dot in the network diagram represents the sample size of the intervention, and the thickness of the line represents the amount of direct evidence between the two interventions. If there was a closed loop in the diagram, the inconsistency test was employed to evaluate consistency between the results of direct comparison and indirect comparison. If no closed loop formed, the consistency model was directly used for analysis. The surface under the cumulative ranking curve (SUCRA) was applied to rank the interventions of each outcome index, with a greater SUCRA value indicating a better the effect of the intervention. A “comparison correction” funnel plot was used to identify a small-sample effect between studies and publication bias in the studies included.

## Results

### Literature Search Results

**Figure 1:**
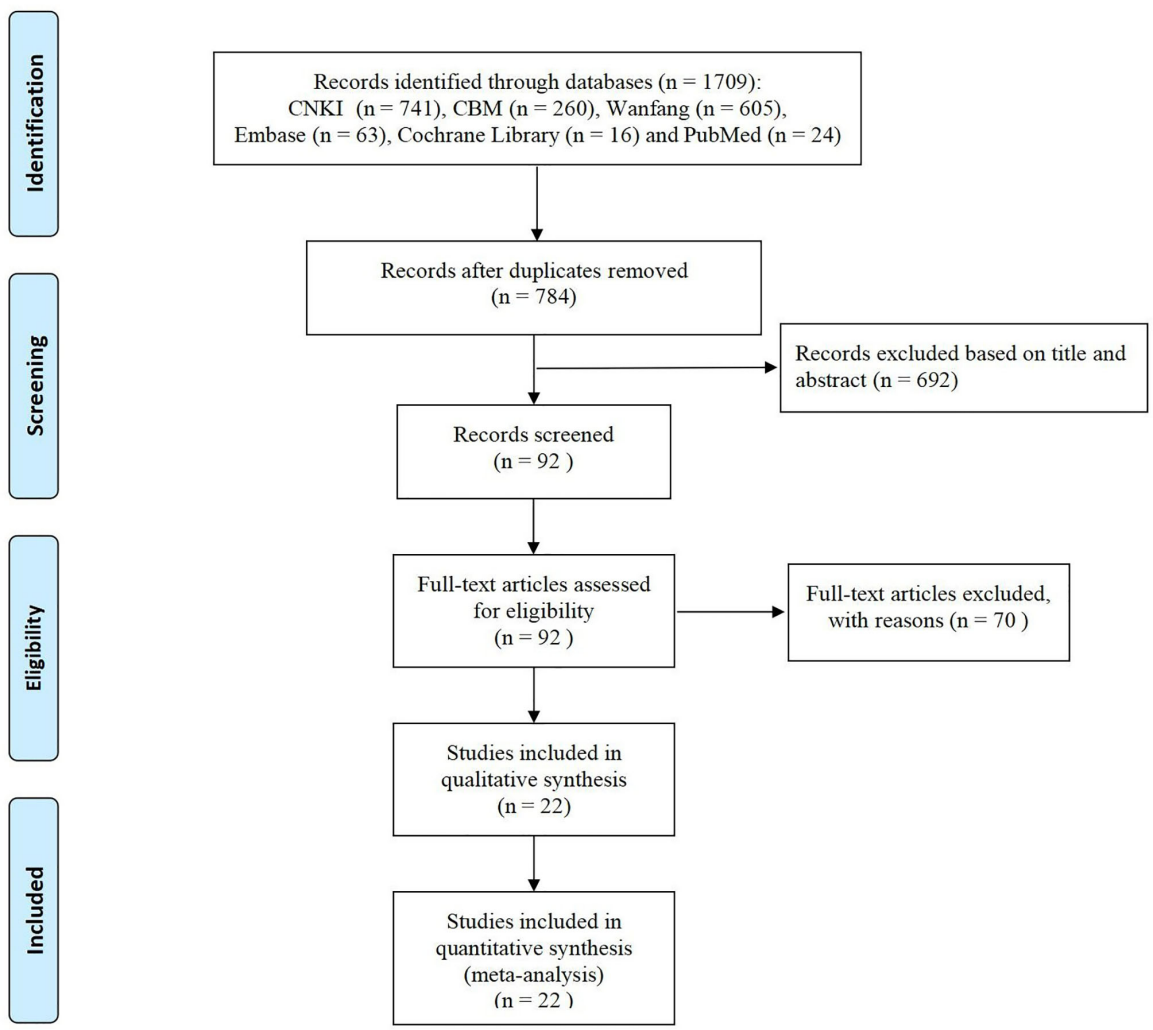
Flow diagram of the study selection process.

We preliminarily searched 1709 relevant studies, and 784 remained after deleting duplicate studies. After reading the titles and abstracts, we screened a total of 92 articles for full-text evaluation. A total of 22 clinical studies (13–34) were included after reading the full texts with reference to our inclusion and exclusion criteria. The process and results of the literature screening are shown in **Figure 1**.

### Basic Characteristics of the Included Studies

A total of 22 studies (13–34) (including 19 RCTs and 3 non-RCTs) were included: 4 three-arm (21, 23, 26, 30) tests and 18 two-arm tests (13–20, 22, 24, 25, 27–29, 31–34). The total sample size was 2016 cases, including 924 cases in the treatment group and 1092 cases in the control group. The treatment course and follow-up time included in the study ranged from 1 to 12 months. Twelve medication measures were involved: calcium, XLGB, bisphosphonate (BP), XLGB+calcium, XLGB+BP, XLGB +BP+calcium, XLGB+vitamin D (VD), VD, BP+calcium, calcium+VD, estrogen+calcium, and XLGB+calcium+VD. The basic information of the included studies is shown in **Table 1**.

**Table 1:** The characteristics of the included studies.

Included studies	Treatment Group 1				Treatment Group 2				Study design	Treatment course (months)
	Treatment	Sample size	Age (years)	Years since menopause (years)	Treatment	Sample size	Age (years)	Years since menopause (years)		
Zhu HM (13)	XLGB+Calcium+VD	61	65.4 ± 6.3	15.5 ± 6.4	Calcium+VD	61	64.9 ± 6.0	15.1 ± 7.2	RCT	12
Zhang H (14)	XLGB+BP+Calcium	28	68.34 ± 3.25	–	–	28	67.64 ± 3.56	–	Non - RCT	12
Wu N (15)	XLGB+BP+Calcium	33	54.2 ± 4.2	–	Calcium	23	55.6 ± 3.9	–	RCT	6
Li L (16)	XLGB+BP+Calcium	34	58.2 ± 0.3	3.5 ± 0.4	BP+Calcium	34	58.5 ± 0.3	3.2 ± 0.5	RCT	6
Ni G (17)	XLGB	35	66.3 ± 5.8	14.8 ± 4.5	XLGB+VD	45	65.1 ± 7.9	16.9 ± 7.2	RCT	2
Wang M (18)	XLGB+VD	45	65.4 ± 7.3	15.6 ± 6.4	BP	45	64.7 ± 8.2	15.2 ± 7.1	RCT	6
Lai F (19)	XLGB+BP	30	63.52	–	BP	30	63.15	–	Non - RCT	3
Dai Y (20)	XLGB+Calcium	53	55.88 ± 5.64	6.89 ± 3.59	Calcium	53	56.73 ± 5.17	7.83 ± 2.63	RCT	1
Zhang X* (21)	XLGB+Calcium	62	–	–	Estrogen+Calcium	66	–	–	RCT	12
					Calcium	50	–	–		
Wu W (22)	XLGB+Calcium	34	55.6 ± 4.3	13.6 ± 3.4	Calcium	34	56.4 ± 4.6	13.8 ± 4.1	RCT	12
Xu M* (23)	XLGB+BP	52	–	–	BP	52	–	–	RCT	6
					XLGB	52	–	–		
Cai C (24)	XLGB+BP	63	58.79 ± 8.82	5.86 ± 1.52	BP	63	59.66 ± 7.34	5.92 ± 1.55	RCT	6
Nie D (25)	XLGB+VD	35	59.8	48.6	VD	35	60.3	49.3	RCT	2
Wu Z* (26)	XLGB+Calcium	38	51.2 ± 3.2	6.3 ± 1.1	Calcium	37	56.3 ± 3.5	6.7 ± 2.0	RCT	1
					XLGB	33	55.1 ± 2.9	6.5 ± 2.3		
Wang Y (27)	XLGB+VD	50	67.81 ± 7.04	13.25 ± 5.62	VD	50	66.74 ± 6.85	13.85 ± 5.19	RCT	2
Li Y (28)	XLGB+Calcium	51	62.2 ± 2.3	9.9 ± 1.4	Calcium	51	61.8 ± 2.5	9.8 ± 1.6	RCT	–
Chen X (29)	XLGB+Calcium+VD	30	56.45 ± 5.33	5.84 ± 4.43	Calcium+VD	30	54.86 ± 5.19	5.66 ± 4.28	RCT	6
Shang Y* (30)	XLGB+Calcium	30	–	–	Estrogen+Calcium	30	–	–	RCT	6
					Calcium	30	–	–		
Liu M (31)	XLGB	34	73.50 ± 12.25	–	Calcium	34	73 ± 12.16	–	RCT	1
Song X (32)	XLGB+Calcium	31	56 ± 3.16	8.32 ± 2.67	Calcium	31	55.21 ± 2.97	7.28 ± 1.95	RCT	6
Zhou J (33)	XLGB+BP+Calcium	63	66.79 ± 8.16	12.29 ± 2.07	BP+Calcium	63	66.72 ± 8.24	13.12 ± 2.15	Non - RCT	6
Zhuang L (34)	XLGB+BP	32	59.78 ± 2.17	–	–	32	60.34 ± 2.35	–	RCT	6

XLGB, Xianling Gubao Capsule; VD, vitamin D; BP, bisphosphonate; RCT, randomized controlled trail. *Three-arm experiment.

### Quality Evaluation of the Included Studies

**Figure 2:**
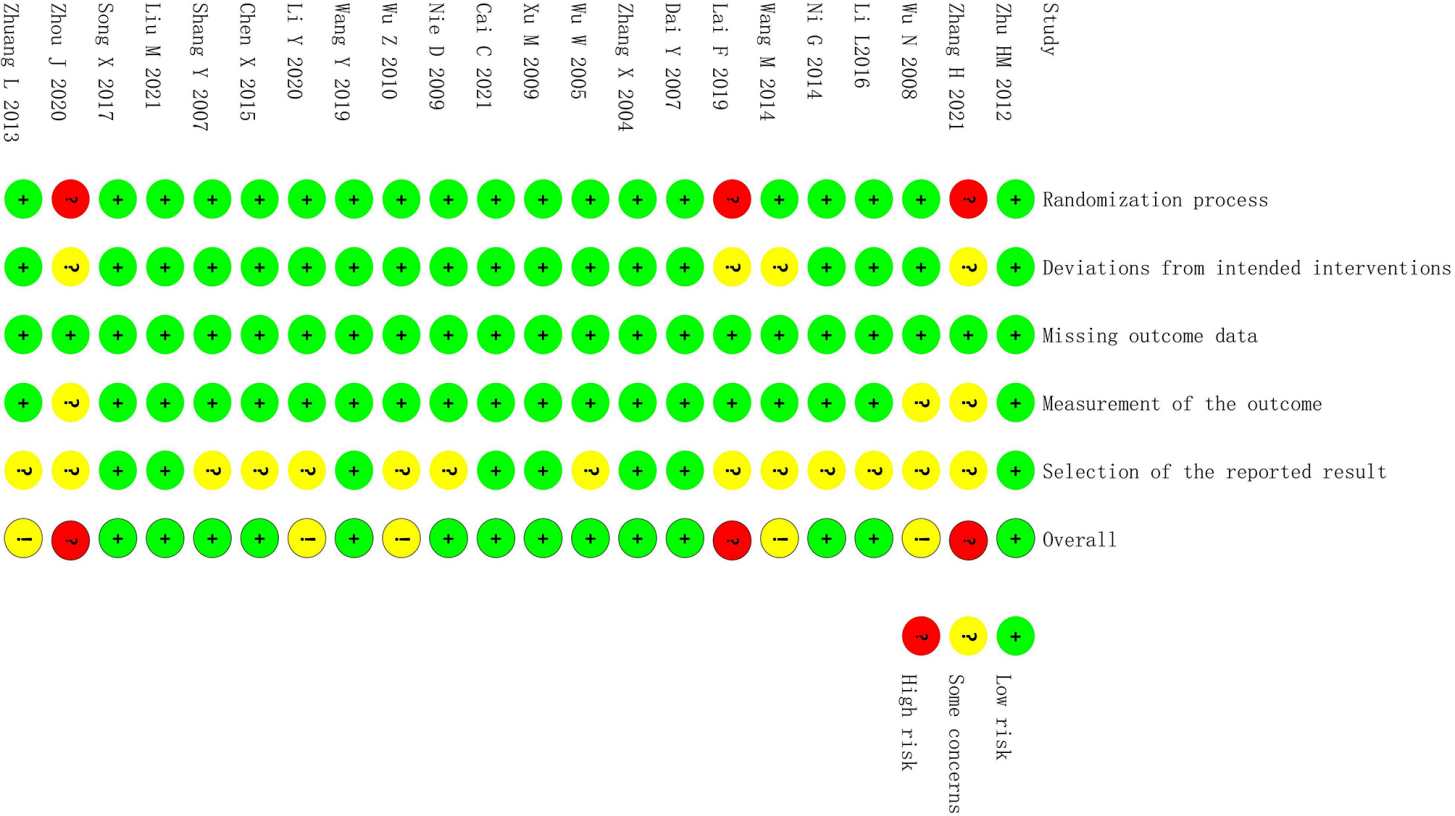
Risk of bias.

Nineteen studies (13, 15–18, 20–32, 34) reported specific methods of random assignment or a description of “random” assignment. In 18 studies (13, 15–17, 20–32, 34), intervention was according to the research protocol, whereas the specific basic treatment was not specified in the other 4 studies (14, 17, 19, 33). The outcome index data of all studies were complete and extractable. Because 3 studies (14, 15, 33) did not implement strict blinding methods, measurement bias may have been present. No selective reports were found. Overall, the quality of the literature included was general, and a more rigorous double-blind trial design is needed. The risk assessment of the included studies is shown in **Figure 2**.

### Traditional Meta-analysis

1) Lumbar BMD (**Table 2**). Compared with calcium alone, XLGB+calcium significantly improved lumbar BMD (MD = 0.13, 95% CI [0.03, 0.22]). In addition, XLGB+VD showed more advantages in improving lumbar BMD compared with VD (MD = 0.09, 95% CI [0.04, 0.14]) or XLGB (MD = -0.09, 95% CI [-0.12, -0.06]) alone. Overall, the combined application of XLGB can improve the BMD of the lumbar spine compared with BP (MD = 0.10, 95% CI [0.07, 0.13]) or BP+calcium (MD = 0.13, 95% CI [0.08, 0.18]).

**Table 2:** Traditional Meta-analysis of Lumbar BMD.

Intervention	No. of studies	I^2^, %	MD (95% CI)	p value
XLGB+Calcium *vs* Calcium	5	96	0.13 (0.03, 0.22)	0.01
XLGB+BP *vs* BP	3	62	0.04 (0.00, 0.08)	0.05
XLGB+Calcium+VD *vs* Calcium+VD	2	98	0.10 (-0.10, 0.30)	0.32
XLGB+BP+Calcium vs BP	1	–	0.10 (0.07, 0.13)	<0.001
XLGB vs XLGB+VD	1	–	-0.09 (-0.12, -0.06)	<0.001
XLGB+VD vs BP	1	–	0.01 (-0.02, 0.03)	0.70
XLGB+VD vs VD	1	–	0.09 (0.04, 0.14)	0.001
BP vs Calcium	1	–	0.10 (0.05, 0.15)	<0.001
XLGB+BP+Calcium vs BP+Calcium	1	–	0.13 (0.08, 0.18)	<0.001

2) Femoral neck BMD (**Table 3**). Compared with VD (MD = 0.03, 95% CI [0.01, 0.06]) or XLGB (MD = -0.09, 95% CI [-0.11, -0.07]) alone, XLGB+VD significantly improved femoral neck the BMD. Compared with calcium alone (MD = 0.17, 95% CI [0.06, 0.29]) or BP+calcium (MD = 0.09, 95% CI [0.03, 0.15]), combined application of XLGB displayed a synergistic role in improving the BMD of the femoral neck.

**Table 3:** Traditional Meta-analysis of femoral neck BMD.

Intervention	No. of studies	I^2^, %	MD (95% CI)	p value
XLGB+Calcium vs Calcium	4	98	0.17 (0.06, 0.29)	0.003
XLGB+BP vs BP	2	78	0.04 (-0.03, 0.10)	0.24
XLGB+Calcium+VD vs Calcium+VD	2	97	0.08 (-0.10, 0.26)	0.38
XLGB+VD vs VD	2	23	0.03 (0.01, 0.06)	0.02
XLGB vs XLGB+VD	1	–	-0.09 (-0.11, -0.07)	<0.001
XLGB+VD vs BP	1	–	0.01 (-0.02, 0.04)	0.44
XLGB vs Calcium	1	–	0.06 (-0.00, 0.12)	0.05
XLGB+BP+Calcium vs BP+Calcium	1	–	0.09 (0.03, 0.15)	0.006

3) ALP (**Table 4**). Compared with calcium alone (MD = 4.26, 95% CI [0.53, 7.98]), XLGB+calcium significantly increased the content of ALP in serum. XLGB+BP+calcium also increased serum ALP compared with BP alone (MD = 2.82, 95% CI [0.89, 4.75]). Nevertheless, XLGB+BP (MD = -2.55, 95% CI [-4.06, -1.04]) or XLGB+BP+calcium (MD = -15.70, 95% CI [-21.51, -9.89]) combination therapy did not necessarily increase the ALP content in serum compared with BP or calcium alone.

**Table 4:** Traditional Meta-analysis of BGP.

Intervention	No. of studies	I^2^, %	MD (95% CI)	p value
XLGB+Calcium *vs* Calcium	4	98	4.26 (0.53, 7.98)	0.03
XLGB+BP+Calcium vs BP+Calcium	2	99	3.23 (-30.54,-37.00)	0.85
XLGB+BP *vs* BP	1	–	-2.55 (-4.06, -1.04)	<0.001
XLGB+Calcium+VD *vs* Calcium+VD	1	–	0.45 (-2.50, 3.40)	0.76
XLGB+BP+Calcium vs BP	1	–	2.82 (0.89, 4.75)	0.004
XLGB+BP+Calcium vs Calcium	1	–	-15.70(-21.51,-9.89)	<0.001

4) BGP (**Table 5**). Compared with calcium alone, XLGB+calcium increased the content of BGP in serum (MD = 3.75, 95% CI [0.59, 6.91]), and XLGB+BP+calcium reduced serum BGP compared with BP+calcium (MD = -24.97, 95% CI [-31.66, -18.28]).

**Table 5:** Traditional Meta-analysis of ALP.

Intervention	No. of studies	I^2^, %	MD (95% CI)	p value
XLGB+Calcium *vs* Calcium	5	100	5.36(-26.04, 36.76)	0.74
XLGB+VD vs BP	1	–	-1.00(-3.03, 1.03)	0.33
XLGB vs Calcium	1	–	3.75 (0.59, 6.91)	0.02
XLGB+BP+Calcium vs BP+Calcium	1	–	-24.97 (-31.66, -18.28)	<0.001

### Network Meta-analysis

#### Network Diagram

**Figure 3:**
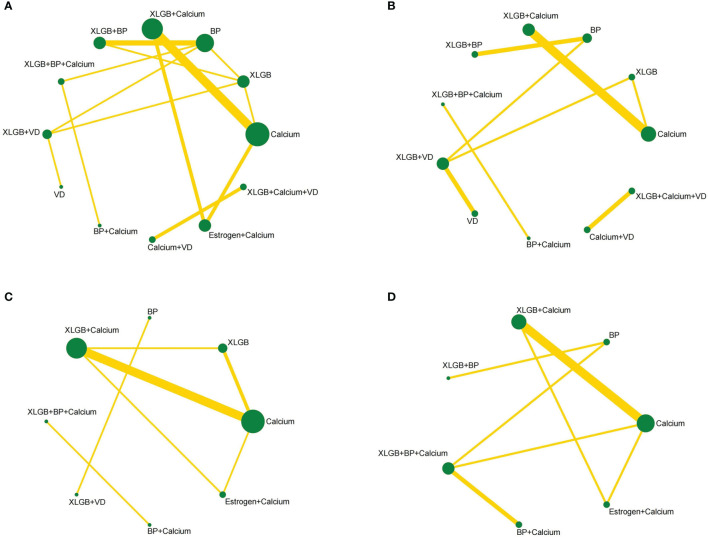
Network diagrams depicting direct evidence used in network meta-analysis. **(A)** Network diagram of lumbar BMD. **(B)** Network diagram of BMD of femoral neck. **(C)** Network diagram of BGP. **(D)** Network diagram of ALP.

This study included 12 interventions, 5 of which involved XLGB. The evidence network of the four outcome indicators is shown in **Figure 3**. The lines in the figure represent interventions with a direct comparison, the line thickness represents the number of studies, and the dot size represents the sample size of the intervention.

#### Inconsistency Test of Closed Loops

The 12 interventions of lumbar BMD formed three closed loops. See **Table 6** for the detection results of the characteristic intercirculation inconsistency factor (IF), 95% CI and intercirculation heterogeneity parameter t^2^ of lumbar BMD. The IF values of the three closed loops were 0.040, 0.085, and 0.127, and the lower limit of the 95% CIs was 0, which indicates that the consistency of each closed loop was good; that is, direct comparison and indirect comparison had little impact on the results of the whole network meta-analysis. The statistical results of the reticular meta-analysis of lumbar BMD were highly reliable. The eight interventions of ALP formed two closed loops. As shown in **Table 6**, the consistency of the two closed loops of ALP was good. The seven interventions of BGP formed a closed loop, with a large lower 95% CI limit (3.40), suggesting inconsistency. Therefore, the results of the network meta-analysis of BGP need to be interpreted carefully.

**Table 6:** Results of the closed-loop inconsistency test.

Outcome indicators	Closed-Loop	IF	95%CI (truncated)	Loop-specific Heterogeneity(t^2^)	z-value	p-value
LBMD	Calcium - XLGB+Calcium - Calcium+VD	0.127	(0.00, 0.36)	0.010	1.058	0.0290
	XLGB - BP - XLGB+VD	0.085	(0.04, 0.13)	0.000	0.023	0.000
	XLGB - BP - XLGB+BP	0.040	(0.00, 0.09)	0.000	0.027	0.142
ALP	Calcium - BP - XLGB+Calcium	71.847	(0.00, 202.01)	741.190	1.082	0.279
	Calcium - XLGB - BP	0.005	(0.00, 129.84)	1233.224	0.000	1.000
BGP	Calcium - BP - XLGB+VD	5.260	(3.40, 7.12)	0.000	5.555	0.000

#### Results of the Network Meta-analysis

In the comparison of lumbar BMD, 45 pairwise comparisons were formed. The results of the network meta-analysis showed obvious advantages for XLGB+calcium in improving lumbar BMD compared with calcium alone (MD = 0.13, 95% CI [0.05, 0.20]), and the difference was statistically significant. In improving femoral neck BMD, 21 pairwise comparisons were formed. The results of the network meta-analysis showed that XLGB+calcium had more advantages than calcium alone (MD = 0.17, 95% CI [0.07, 0.27]). In terms of improving the contents of serum ALP and BGP, the results of the network meta-analysis did not suggest a pairwise comparison with a significant difference. The results of the network meta-analysis of the four outcome indicators concerned in this study are shown in **Tables 7**–**10**.

**Table 7:** Network meta-analysis of lumbar BMD.

XLGB+BP+Calcium									
0.06 (-0.13, 0.25)	XLGB+BP								
0.07 (-0.14, 0.28)	0.00 (-0.15, 0.16)	XLGB+VD							
0.10 (-0.17, 0.38)	0.04 (-0.19, 0.27)	0.04 (-0.19, 0.26)	XLGB + Calcium						
0.10 (-0.06, 0.26)	0.04 (-0.06, 0.13)	0.03 (-0.10, 0.16)	-0.00 (-0.23, 0.22)	BP					
0.13 (-0.16, 0.42)	0.07 (-0.17, 0.31)	0.06 (-0.18, 0.30)	0.03 (-0.08, 0.14)	0.03 (-0.20, 0.27)	Estrogen + Calcium				
0.13 (-0.04, 0.30)	0.07 (-0.19, 0.32)	0.06 (-0.21, 0.33)	0.03 (-0.30, 0.35)	0.03 (-0.21, 0.27)	-0.00 (-0.34, 0.33)	BP+Calcium			
0.13 (-0.07, 0.34)	0.07 (-0.07, 0.20)	0.06 (-0.07, 0.19)	0.03 (-0.16, 0.21)	0.03 (-0.09, 0.16)	0.00 (-0.20, 0.20)	0.00 (-0.27, 0.27)	XLGB		
0.16 (-0.11, 0.43)	0.09 (-0.13, 0.32)	0.09 (-0.08, 0.26)	0.05 (-0.23, 0.34)	0.06 (-0.16, 0.27)	0.03 (-0.27, 0.32)	0.03 (-0.29, 0.35)	0.03 (-0.19, 0.24)	VD	
0.23 (-0.04, 0.50)	0.17 (-0.05, 0.38)	0.16 (-0.05, 0.37)	0.13 (0.05, 0.20)	0.13 (-0.08, 0.34)	0.10 (-0.01, 0.21)	0.10 (-0.21, 0.42)	0.10 (-0.07, 0.27)	0.07 (-0.20, 0.34)	Calcium

**Table 8:** Network meta-analysis of femoral neck BMD.

XLGB+Calcium						
0.00 (-0.38, 0.38)	XLGB+BP					
0.02 (-0.27, 0.32)	0.02 (-0.21, 0.26)	XLGB+VD				
0.04 (-0.31, 0.39)	0.04 (-0.10, 0.18)	0.01 (-0.18, 0.21)	BP			
0.06 (-0.27, 0.38)	0.06 (-0.22, 0.33)	0.03 (-0.10, 0.17)	0.02 (-0.22, 0.26)	VD		
0.11 (-0.11, 0.33)	0.11 (-0.19, 0.42)	0.09 (-0.10, 0.28)	0.08 (-0.20, 0.35)	0.06 (-0.18, 0.29)	XLGB	
0.17 (0.07, 0.27)	0.17 (-0.19, 0.54)	0.15 (-0.13, 0.42)	0.14 (-0.20, 0.47)	0.11 (-0.19, 0.42)	0.06 (-0.14, 0.26)	Calcium

**Table 9:** Network meta-analysis of ALP.

XLGB+Calcium			
2.76 (-61.02, 66.54)	XLGB		
5.44 (-33.30, 44.18)	2.68 (-55.84, 61.19)	Calcium	
14.99 (-63.45, 93.43)	12.23 (-83.43, 107.89)	9.55 (-68.90, 88.01)	Estrogen+Calcium

**Table 10:** Network meta-analysis of BGP.

XLGB+Calcium						
2.17 (-20.15, 24.49)	Estrogen+Calcium					
4.82 (-7.62, 17.25)	2.65 (-19.67, 24.97)	Calcium				
19.90 (-7.98, 47.78)	17.73 (-15.75, 51.21)	15.08 (-9.88, 40.04)	XLGB+BP+Calcium			
22.46 (-14.53, 59.45)	20.29 (-21.08, 61.67)	17.65 (-17.19, 52.48)	2.56 (-22.09, 27.22)	BP		
24.93 (-19.45, 69.31)	22.76 (-25.34, 70.86)	20.12 (-22.49, 62.72)	5.03 (-29.82, 39.88)	2.47 (-22.27, 27.21)	XLGB+BP	
23.33 (-9.63, 56.28)	21.16 (-16.66, 58.97)	18.51 (-12.02, 49.04)	3.43 (-14.25, 21.10)	0.86 (-29.46, 31.19)	-1.60 (-40.67, 37.46)	BP+Calcium

#### SUCRA Probability Ranking

The best SUCRA probability rankings of the improvement effects of different interventions on the four outcome indicators are provided in **Table 11**. In terms of improving lumbar BMD, the best three interventions were XLGB+ BP+calcium (83.7%), XLGB+BP (68.5.7%) and XLGB+VD (67.1%). The three best interventions to improve femoral neck BMD were XLGB+calcium (68.0%), XLGB+BP (67.8%) and XLGB+VD (63.0%). The best interventions to increase the serum ALP content in were XLGB+calcium (59.5%), XLGB (52.6%) and calcium (47.8%). The best measures to improve the serum BGP content were XLGB+calcium (82.1%), estrogen+calcium (73.5%) and calcium (67.7%).

**Table 11:** Rank of efficacy of included treatments.

Outcome indicator Treatment methods	BMD of lumbar vertebra		BMD of femoral neck		ALP		BGP	
	SUCRA value	Rank	SUCRA value	Rank	SUCRA value	Rank	SUCRA value	Rank
Calcium	11.6	10	16.9	7	47.8	3	67.7	3
XLGB	40.2	8	31.7	6	52.6	2	–	–
BP	51.9	5	54.4	4	–	–	32.8	5
XLGB + Calcium	54.8	4	68.0	1	59.5	1	82.1	1
XLGB + BP	68.5	2	67.8	2	–	–	28.5	7
XLGB + BP + Calcium	83.7	1	–	–	–	–	37.1	4
XLGB + VD	67.1	3	63.0	3	–	–	–	–
VD	34.9	9	48.3	5	–	–	–	–
BP + Calcium	42.1	7	–	–	–	–	28.4	6
Calcium + VD	–	–	–	–	–	–	–	–
Estrogen + Calcium	45.1	6	–	–	40.1	4	73.5	2
XLGB + Calcium + VD	–	–	–	–	–	–	–	–

#### Small-Sample Effect Evaluation

**Figure 4:**
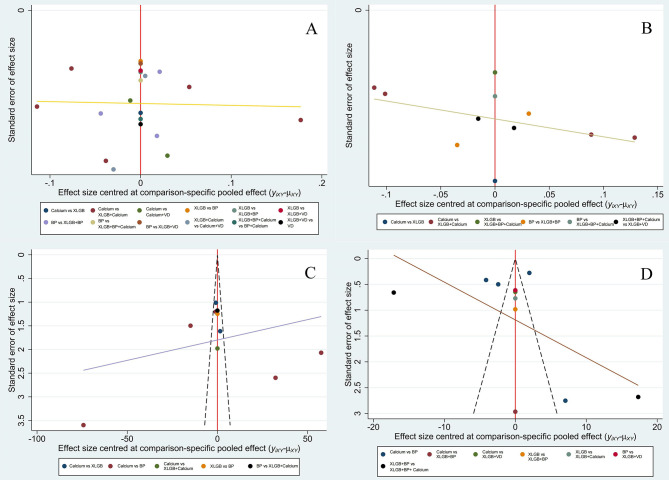
Comparison-adjusted funnel plot of lumbar BMD **(A)**, BMD of the femoral neck **(B)**, BGP **(C)** and ALP **(D)**.

A funnel plot was drawn to evaluate a small-sample effect for the four outcome indicators in this study (**Figure 4**). Different colors in the funnel chart indicate different intervention measures. We found that most dots in the funnel chart of BMD of the lumbar spine and femoral neck were roughly symmetrically distributed on both sides of the vertical line with X = 0, which suggests no publication bias for these two outcome indicators. For ALP and BGP, a high degree of publication bias and a small sample effect were suggested.

## Discussion

This study evaluated the efficacy of XLGB and other combined therapies for the treatment of PMOP by network meta-analysis. The results show that the combined application of XLGB can well treat PMOP. Osteoporosis is a systemic bone disease characterized by reduced bone mass and degeneration of bone microstructure, which leads to increased bone fragility and easy fracture (1). Among the types of osteoporosis, PMOP has a high incidence rate (3). XLGB is a Chinese patent medicine widely used for the treatment of osteoporosis (35–37). Due to the lack of comparisons between XLGB and other drug regimens in the treatment of PMOP, this study ranked the clinical efficacy of XLGB-related drug regimens in PMOP treatment with the help of network meta-analysis to provide an evidence-based foundation for its clinical use.

Traditional meta-analysis is a direct comparison between intervention measures, which can most intuitively compare differences in efficacy among drug regimens. In terms of improving lumbar BMD and femoral neck BMD (13, 14, 17, 18, 20–25, 27–34), combined application of XLGB can improve lumbar BMD and femoral neck BMD compared with calcium and VD alone, and XLGB+BP+calcium showed more advantages than BP alone. Furthermore, combined application of XLGB on the basis of BP+calcium had a good clinical effect on improving lumbar BMD. XLGB+calcium exhibited more advantages than calcium alone in increasing the content of serum BGP, and XLGB+BP+calcium had better clinical efficacy than BP alone (13–16, 20–22, 24, 28, 33). Moreover, XLGB showed better clinical efficacy than calcium in improving serum ALP content.

Network meta-analysis was used to examine differences among interventions through indirect comparisons. The results of this study show that XLGB+BP+calcium is most likely the optimal treatment for improving lumbar BMD but that XLGB+calcium is the best combination regimen for improving femoral neck BMD and increasing contents of serum BGP and ALP. Combining the results of this traditional meta-analysis and network meta-analysis, compared with the schemes of VD, BP or calcium alone, combined application of XLGB showed better clinical efficacy in improving BMD and serum BGP and ALP. In traditional Chinese medicine, orthopedic diseases such as osteoporosis are believed to be the manifestation of kidney deficiency. The kidney governs bone and generates marrow, which should be used to tonify the kidney and strengthen *Yang* (38, 39). Deficiency of the kidney and spleen is the root cause of the disease, and blood stasis is the promoting factor. Blood stasis further aggravates injury to the spleen and kidney and then accelerates the occurrence of osteoporosis (39). XLGB, which is mainly composed of *Epimedium* supplemented with *Dipsacus*, *Salvia miltiorrhiza*, *Rehmannia glutinosa* and *Anemarrhena asphodeloides*, has the effects of nourishing the liver and kidney, activating blood circulation and dredging collaterals, and strengthening tendons and bones (40). Previous studies have shown that XLGB combined with calcium or BP has a better clinical effect in the treatment of osteoporosis (7, 41), which is consistent with our conclusions. In addition, theoretical studies have shown that XLGB inhibits osteoclast activity and promotes osteoblast differentiation by regulating OPG/RANK/RANKL (42). Animal experiments have shown that XLGB increases the contents of Ca and P in rat serum, reduces the contents of ALP, OCN and DPD in serum, and increases the BMD of vertebrae as well as the femur and tibia (43). The above results provide a theoretical basis for using XLGB to treat PMOP.

This study has the following limitations. 1) Due to the lack of direct comparison evidence for each outcome index, it was not possible to rank the probability of individual interventions. 2) Most of the included studies were small-sample studies, reducing the statistical efficiency of tour analysis. None of the studies provided strict sample-size estimation standards, which may affect authenticity. 3) We also detected statistical heterogeneity and publication bias, and a pharmacoeconomic evaluation was not included. Furthermore, the great differences in the course of treatment and follow-up time included in the literature may also affect the credibility of the results. Therefore, the conclusions of this study need to be applied in combination with clinical practice. 4) Due to the limitations of this study, further animal or cell experiments are needed to verify the results to provide a more experimental or clinical basis.

## Conclusion

The results of this network meta-analysis show that combined application of XLGB can effectively improve BMD and serum BGP and ALP compared to calcium, VD or BP alone. In the future, multicenter, large-sample and double-blind clinical RCTs need to be carried out to supplement and validate the results of this study.

## Data Availability Statement

The original contributions presented in the study are included in the article/**Supplementary Material**. Further inquiries can be directed to the corresponding authors.

## Author Contributions

M-hL and J-lZ conceptualized the research question. M-hL and J-lZ participated in drafting and writing the review. N-jX, XX, W-xF, and J-lZ participated in the formulation of retrieval strategies, data acquisition, data analysis, and quality assessment. M-hL, XX, and J-lZ participated in the drawing of tables and figures. Z-pL and L-fZ participated in critical revision of the manuscript. All authors contributed to the research and approved the final manuscript.

## Funding

This work was supported by the National Natural Science Foundation of China (No.82004383, No. 81974574), the National key research and development program (2021YFC1712804), the Project of Administration of Traditional Chinese Medicine of Guangdong Province (No.20201129), the Project of Guangdong Provincial Department of Finance (No. [2014]157, No. [2018]8), the Medical Science Research Foundation of Guangdong Province (No.A2020105, No.B2019091), the Science and Technology Planning Project of Guangzhou (No. 202102010273) and the Science and Technology Research Project of Guangdong Provincial Hospital of Chinese Medicine (No.YN2019ML08, YN2015MS15).

## Conflict of Interest

The authors declare that the research was conducted in the absence of any commercial or financial relationships that could be construed as a potential conflict of interest.

## Publisher’s Note

All claims expressed in this article are solely those of the authors and do not necessarily represent those of their affiliated organizations, or those of the publisher, the editors and the reviewers. Any product that may be evaluated in this article, or claim that may be made by its manufacturer, is not guaranteed or endorsed by the publisher.
