# Supplementary material for: Comparative Efficacy of Xianling Gubao Capsules in Improving Bone Mineral Density in Postmenopausal Osteoporosis: A Network Meta-Analysis
Source: Front Endocrinol (Lausanne). 2022 Feb 18;13:839885. doi: 10.3389/fendo.2022.839885 (PMC8895757; doi:10.3389/fendo.2022.839885)
Supplement: Supplementary file 1 [file DataSheet_1.docx]

**Supplementary material 1 The retrieval strategy of each database.**

**CNKI**

"Xianling Gubao"[TKA] AND "osteoporosis"[TKA]

**SinoMed**

"Xianling Gubao"[Title] AND "osteoporosis"[Title]

**Wanfang database**

"Xianling Gubao"[Title or Key words] AND "osteoporosis"[Title or Key words]

**PubMed**

("xianling gubao"[Title/Abstract] OR "Xianlinggubao"[Title/Abstract] OR "XLGB"[Title/Abstract]) AND ("osteoporosis, postmenopausal"[MeSH Terms] OR "osteoporosis"[MeSH Terms] OR "osteoporosis, postmenopausal"[MeSH Terms] OR "osteoporosis, postmenopausal"[MeSH Terms] OR (("primaries"[All Fields] OR "primary"[All Fields]) AND ("osteoporosis, postmenopausal"[MeSH Terms] OR "osteoporosis"[MeSH Terms])))

**Cochrane Library**

#1 (xianling gubao):ti,ab,kw OR (Xianlinggubao):ti,ab,kw OR (XLGB):ti,ab,kw

#2 (Osteoporosis):ti,ab,kw OR (Postmenopausal osteoporosis):ti,ab,kw OR (Post-Menopausal Osteoporosis):ti,ab,kw OR (Primary osteoporosis):ti,ab,kw

#3 #1 AND #2

**Embase**

#1 'xianling gubao':ab,ti OR xianlinggubao:ab,ti OR xlgb:ab,ti

#2 osteoporosis:ab,ti OR 'postmenopausal osteoporosis':ab,ti OR 'post-menopausal osteoporosis':ab,ti OR 'primary osteoporosis':ab,ti

#3 #1 AND #2
